# Personality and Motivation to Comply With COVID-19 Protective Measures in Germany

**DOI:** 10.3389/fpsyg.2022.893881

**Published:** 2022-06-13

**Authors:** Kai Kaspar, Laura Nordmeyer

**Affiliations:** Department of Psychology, University of Cologne, Cologne, Germany

**Keywords:** COVID-19, protective measures, demographic variables, risk perception, personality, conspiracy mentality, locus of control, general affect

## Abstract

The COVID-19 pandemic strains the healthcare systems, economy, education, and social life. Governments took several protective measures and formulated behavioral guidelines to prevent individual diseases and the collapse of healthcare systems. However, individual differences in the extent of compliance with the measures are apparent. To shed more light on this issue, the present correlational study examined the joint relation of several personal characteristics to people's motivation to comply with seven protective measures. Personal characteristics included age, gender, risk perception, the Big Five, the Dark Triad, conspiracy mentality, perceived locus of control, and general affect. Protective measures included social distancing, hygiene rules, wearing face masks, using a contact-tracing app, sharing one's infection status *via* the app, reducing physical contacts, and vaccinations. The study ran from 10 November 2020 to 29 December 2020. Based on a sample of 1,007 German-speaking participants, bivariate correlations and multiple regression analyses showed that personal characteristics are significantly linked to the motivation to comply with these measures. However, general affect, control beliefs, and basic personality traits play only a minor role. Age and gender showed some significant associations with protective measures. In contrast, protection motivation factors, in terms of perceived severity of and vulnerability to infection, and conspiracy mentality appear to be the major correlates of adopting protective behavior. The absolute motivation to comply with the measures also shows that hygiene rules and wearing face masks receive a higher average agreement than more personally intrusive measures such as physical contact restrictions and vaccinations. These results highlight that factors that are relevant to some measures may be irrelevant to other measures. Differences in people's personal characteristics should be considered in the design and communication of measures to support social acceptance and effectiveness. In this context, cognitive variables, which can be addressed by communication and education directly, seem to be more important than general affect and relatively time-invariant personality traits.

## Introduction

The COVID-19 disease, caused by the novel coronavirus SARS-CoV-2, has been disrupting the world and putting society in a state of emergency with both physical and psychological consequences (Talevi et al., 2020). By 11 March 2020, more than 100 countries and more than 118,000 individuals had already been affected, leading the World Health Organization (WHO) to declare COVID-19 a global pandemic (WHO, 2020). This pandemic extensively strains healthcare systems, economy, education, and social life. To slow down the spread of the virus and prevent a collapse of healthcare systems, governmental measures were introduced that led to significant changes in private and public life. Measures include repeated lockdowns, curfews, closures of schools, kindergartens, stores, and borders, as well as home-based work and a ban on social gatherings (Khurshid et al., 2020). In addition, behavioral guidelines were formulated. To minimize transmission risks, the WHO recommended physical distancing, use of masks, proper ventilation of rooms, avoidance of crowds, hand hygiene, and appropriate sneezing and coughing (WHO, 2021). However, not everyone adheres to these measures to the same degree and behavioral differences can be observed (IGHI, 2020).

The question arises as to what motivates people to comply with COVID-19 protective measures. Several studies have already addressed this question, focusing on the role of person-related characteristics. Indeed, associations between adherence to guidelines and several personality traits have been reported. For example, conscientiousness, openness, and positive attitudes (Bogg and Milad, 2020), but also factors such as psychopathy and meanness (Blagov, 2021) were among the relevant variables. However, personal characteristics have not been comprehensively evaluated in the context of compliance with COVID-19 protective measures. As for the measures, they were either grouped together and were reported rather nonspecifically, or only a few specific measures were addressed. For example, surveys asked about overall compliance with measures without specifying them (e.g., Zajenkowski et al., 2020) or different measures were combined to composite values (e.g., mean value across all measures) in the central regression analyses (e.g., Dohle et al., 2020) and path analyses (e.g., Bogg and Milad, 2020), preventing a more nuanced picture. Other studies limited their scope to a few specific domains like social distancing (e.g., Abdelrahman, 2020; Carvalho et al., 2020; Götz et al., 2020). In terms of personal characteristics, the focus of previous studies has been rather narrow, that is, they focused on one or a few personality constructs such as the Big Five (see Section Big Five Personality Traits) or the Dark Triad (see Section Dark Triad Personality Traits) (Abdelrahman, 2020; Asselmann et al., 2020; Aschwanden et al., 2021; Blagov, 2021). We would like to emphasize that all these studies have provided very valuable insights. The present study aims to fill the empirical gap outlined above. Therefore, a comprehensive set of personal characteristics is included in this study to measure their joint contribution to people's motivation to comply with various COVID-19 protective measures.

### COVID-19 Protective Measures

This study focuses on seven key measures to combat the pandemic. Three measures refer to the standard measures formulated by German health and governmental authorities (“AHA-rules”): social distancing in terms of keeping a minimum distance of 1.5 m from other persons in public (i.e., distance rule), performing hygiene (i.e., appropriate coughing and sneezing as well as washing hands regularly and thoroughly), and wearing face masks in certain areas of public life (BZgA, 2021). The fourth measure concerns the restriction of physical contacts, which has been implemented on a large scale locally and globally (e.g., Bönisch et al., 2020; Lai et al., 2021). Complementing this, the fifth measure is a technical approach to the pandemic, namely the active use of a contact-tracing app. The German version of this app, which is the focus here, informs its users about critical contacts with infected individuals. This measure is intended to detect and interrupt chains of infection. However, infected people decide for themselves whether they want to share their infection status *via* the app. Therefore, besides just using the app, sharing one's infection status is an important sixth measure. It should be noted that these app-related measures are less preventive than other measures because neither using the app nor sharing one's infection status directly helps prevent a COVID-19 infection, but they are helpful to others and to combat the pandemic on a broad societal level (cf. Kaspar, 2020). Finally, the seventh measure is vaccination against the coronavirus. Mass vaccination campaigns are taking place around the world and the relative effectiveness of available vaccinations has been confirmed (e.g., Dagan et al., 2021), but it also depends on virus variants (e.g., Lopez Bernal et al., 2021).

### What Role Do Personal Characteristics Play in COVID-19 Protective Measures?

Previous research and several health theories highlight the critical role of personal characteristics when it comes to the motivation to comply with COVID-19 measures. For example, the Health Belief Model explains and predicts various health behaviors such as influenza vaccination, risky sexual behaviors, and cancer prevention. The model postulates that in addition to individual belief components such as perceived vulnerability to and severity of disease, personal characteristics, including age, gender, and personality traits, act as modifying factors shaping individual beliefs and behavior (Champion and Skinner, 2008). Also, the Protection Motivation Theory focuses on cognitive aspects to explain health-related behaviors (Rogers, 1983). Protection motivation (i.e., the motivation to perform health-beneficial behaviors) is formed through coping appraisal and threat appraisal processes, including evaluations of maladaptive and adaptive behaviors. Accordingly, threat appraisal considers the rewards of not performing the recommended behavior as well as the severity of and vulnerability to negative consequences of performing maladaptive behavior. Coping appraisal evaluates perceived self-efficacy and response efficacy of the adaptive behavior, as well as response costs. The applicability of this theory to COVID-19 measures has been demonstrated (Kaspar, 2020). But importantly, the core components of the theory are additionally complemented by various influential sources of information, which include personal characteristics (Rogers, 1983; Floyd et al., 2000). Similarly, the Theory of Reasoned Action and the Theory of Planned Behavior aim to predict individual behavior, including health behavior. In addition to the main components – attitude, subjective norm, and perceived control – demographic variables, personality traits, and other individual differences are considered as factors influencing behavioral motivation (Montaño and Kasprzyk, 2008).

**Figure 1:**
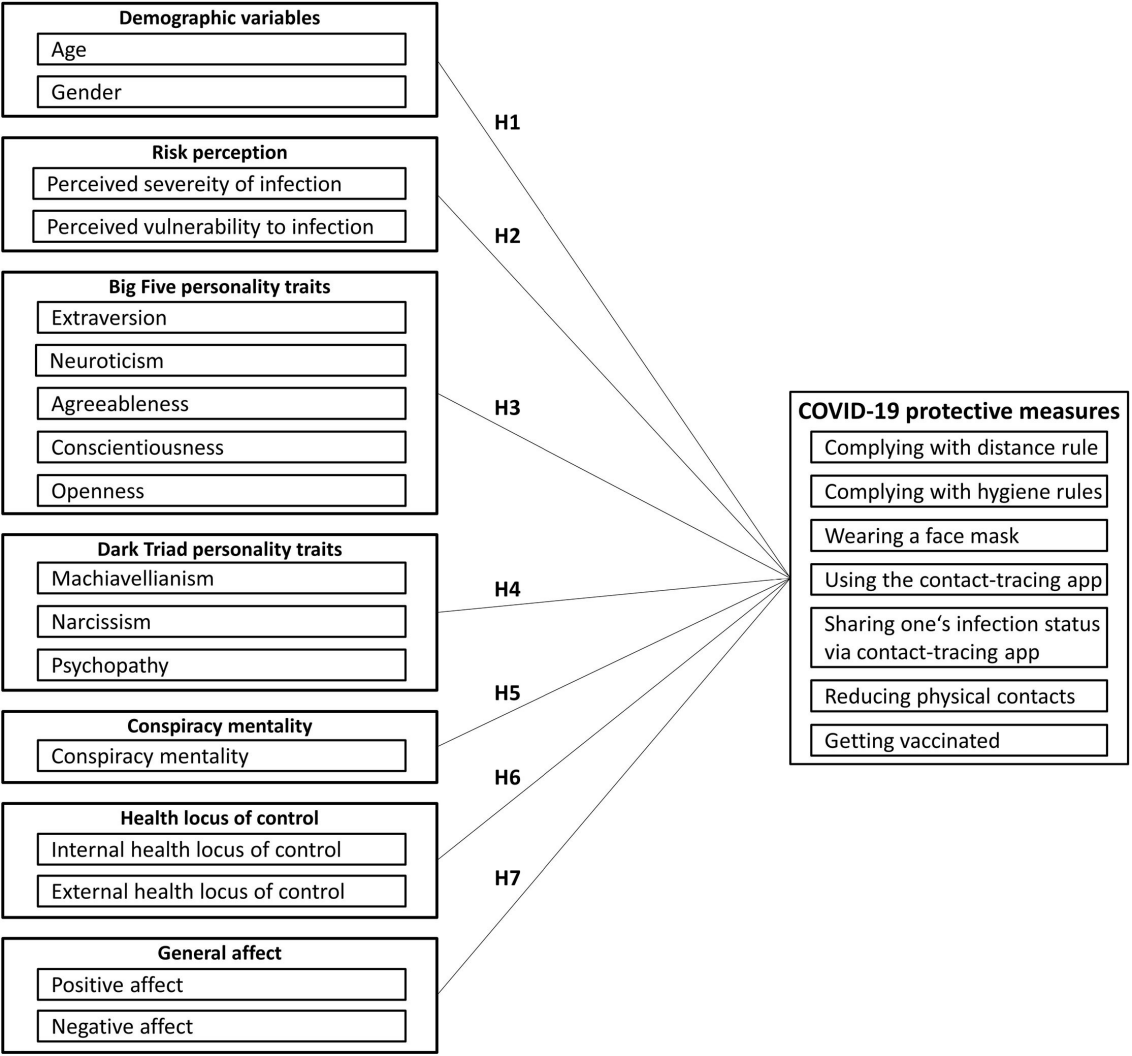
Research model of the present study.

Many of these theories have two things in common: first, they emphasize behavioral motivation as the key outcome variable determining subsequent health-related behavior. Indeed, motivational psychology describes motivation as an internal process that determines the choice, strength, persistence, and actualization of behavior (Becker-Carus and Wendt, 2017), and some authors argue that behavioral motivation is the most reliable predictor of actual behavior (Montaño and Kasprzyk, 2008). Second, personal characteristics, such as personality traits, are included but are not the focus, as they are considered marginal variables that may play an additional or moderating role in behavioral intention. In contrast, the present study shifts the research focus to these personal characteristics. In general, many models in the field of motivation psychology emphasize that the current motivation of a person to strive for a certain goal (such as preventing an infection with the coronavirus) is modulated by personal and situational factors (cf. Heckhausen and Heckhausen, 2006). In addition, according to the accentuation hypothesis formulated by Caspi and Moffitt (1993), personal characteristics “are accentuated when environmental events disrupt previously existing social equilibria” (p. 247) and that they “should predict behavior best in novel, ambiguous, and uncertain circumstances” (p. 267). This description fits very well the overall societal and unpredictable changes brought about by the pandemic, with their impact on the (health) behavior of each individual. Therefore, a significant role of personal characteristics can be assumed when it comes to COVID-19 protective measures. Based on previous research, we propose a research model encompassing several personal characteristics that may jointly contribute to people's motivation to comply with COVID-19 measures. As shown in Figure 1, we consider demographic variables (age and gender), individual risk perception (perceived severity of and vulnerability to infection), general personality traits (Big Five and Dark Triad), individual conspiracy mentality, health-related locus of control beliefs, and general positive and negative affect. The role of these variables in the context of COVID-19 measures and our specific hypotheses are presented in the following section.

### State of Research and Hypotheses

#### Demographic Variables: Age and Gender

Age is a critical variable in the context of the current pandemic as older people are at a higher risk for severe COVID-19 disease (Jordan et al., 2020). Additionally, previous research on COVID-19 found age- and gender-specific relations in compliance with associated measures. For example, both the elderly and women were more likely to consider COVID-19 harmful (Zettler et al., 2022) and reported greater compliance with recommended behavioral measures (Brouard et al., 2020; Zettler et al., 2022). Men and younger people reported lower acceptance and adoption of protective measures (Abdelrahman, 2020; Dohle et al., 2020). However, meta-analytic findings show that fewer women than men stated that they would get vaccinated (Zintel et al., 2022). One reason might be that even before the current COVID-19 pandemic and related vaccines, women typically reported more side effects after vaccinations than men (for a review, see Flanagan et al., 2017). In contrast, age and gender were not related to motivation to use a contract-tracing app and to share one's infection status through the app (Kaspar, 2020). Hence, we hypothesized:

**H1a:** Age is positively related to people's motivation to comply with the distance rule, the hygiene rules, wearing face masks, reducing physical contacts, and vaccination.

**H1b:** Compared to men, women show higher motivation to comply with the distance rule, the hygiene rules, wearing face masks, and reducing physical contacts, but lower motivation to get vaccinated.

#### Risk Perception: Perceived Severity of and Vulnerability to Infection

The Health Belief Model and the Protection Motivation Theory suggest that perceived severity of the infection and perceived vulnerability to infection are relevant in the context of health-related behavior, as they shape perceived threat (Floyd et al., 2000; Champion and Skinner, 2008). Accordingly, higher threat appraisal should increase the likelihood of engaging in protective measures against COVID-19. Indeed, concerning bivariate correlations, both perceived severity and vulnerability showed a positive association with people's motivation for social distancing, using a contact-tracing app and sharing one's infection status *via* the app (Kaspar, 2020). In general, research suggests that there is a positive relation between perceived vulnerability to and the severity of coronavirus infections and compliance with different protective measures (e.g., Berg and Lin, 2020; Bruine de Bruin and Bennett, 2020; Harper et al., 2021). We hypothesized:

**H2:** Perceived severity of the infection and perceived vulnerability to infection are positively related to motivation to comply with all seven COVID-19 measures.

#### Big Five Personality Traits

The Big Five traits are considered the basic dimensions of personality covering a large range of personality facets (Costa and McCrae, 1992), and expert ratings indicate substantial differences in the relative importance of cognitive, affective, and behavioral components of each trait (Pytlik Zillig et al., 2002). The Big Five comprise extraversion (being outgoing and energetic), neuroticism (being nervous and sensitive), agreeableness (being trustful and compassionate), conscientiousness (being organized and efficient), and openness to experience (being creative and curious). A large body of research identified relationships between the Big Five and a wide variety of health behaviors. There are associations between the Big Five and both health-risking and health-promoting behaviors, such as smoking and drug use (Bogg and Roberts, 2004), healthy eating (Raynor and Levine, 2009), the use of health services (Cuijpers et al., 2010), and preventive medical check-ups (Aschwanden et al., 2019). Several studies have already linked Big Five personality traits to COVID-19 measures, revealing rather mixed results. For example, positive relations were found between conscientiousness, agreeableness, openness, and extraversion and adherence to some, but not all recommended protective measures; notably no correlation has been found between extraversion and social distancing (Aschwanden et al., 2021). Carvalho et al. (2020) found that individuals who prefer social distancing to washing hands have lower extraversion scores. Other findings suggest a negative relation between extraversion and general rule-following and taking health precautions (Clark et al., 2020). Similarly mixed results were found with regard to neuroticism. Neuroticism was related to reduced precautions to avoid coronavirus infection (Aschwanden et al., 2021), whereas neuroticism was positively associated with social distancing (Abdelrahman, 2020) and the willingness to reduce contacts by staying at home (Götz et al., 2020) in other studies. Moreover, the role of the Big Five traits regarding the use of contact-tracing apps has not yet been explored. Hence, it seems evident that the Big Five traits are associated with health-related behavior in general as well as in the context of COVID-19. However, further evidence on the relationship between the Big Five traits and various protective measures is needed. Given previous mixed results, we formulated an undirected hypothesis:

**H3:** The Big Five personality traits are related to people's motivation to comply with all seven COVID-19 measures.

#### Dark Triad Personality Traits

In addition to normal range traits, socially aversive traits can also be a relevant factor, namely Machiavellianism (i.e., high self-interest and tendencies toward manipulation, deception, and exploitation of others, including a cynical and instrumental perspective on social interaction), subclinical narcissism (i.e., expressing dominance and superiority along with feelings of grandiosity and entitlement), and subclinical psychopathy (i.e., high impulsivity and thrill-seeking along with anxiety, low empathy, and anti-social behavior) (Paulhus and Williams, 2002). Compliance with COVID-19 measures is not only aimed at protecting oneself, but also at protecting other individuals. A high level of willingness of everyone to comply with the measures is crucial for their effectiveness (BPA, 2020). This very collective idea may not appeal to people with socially aversive characteristics, especially because of their associations with antisocial tendencies (Blagov, 2021). Regarding health, Dark Triad traits served as predictors for protective health behaviors in some studies. They found strong support for a negative relationship between high Dark Triad trait scores and compliance with COVID-19 recommendations such as general governmental restrictions (Zajenkowski et al., 2020), general recommendations and rules to fight COVID-19 (Zettler et al., 2022), and social distancing and hygiene measures (Blagov, 2021). Hence, we hypothesized:

**H4:** The Dark Triad traits are negatively related to people's motivation to comply with all seven COVID-19 measures.

#### Conspiracy Mentality

Conspiracy mentality describes the general tendency to disbelieve common explanations of important social events and phenomena, and instead embrace conspiracy theories that blame other powerful individuals with malicious intentions for these events (Bruder et al., 2013). Even before the pandemic, cross-cultural studies found that higher belief in conspiracy theories was associated with stronger anti-vaccination attitudes (Hornsey et al., 2018). Some conspiracy theories and beliefs have emerged in the context of COVID-19 (cf. Juanchich et al., 2021), and some authors have reported a negative relation between COVID-19 conspiracy beliefs and health-protective behaviors (Allington et al., 2021). Romer and Jamieson (2020) found that specific conspiracy theories related to COVID-19 were stable from March to July 2020 in a U.S. sample. Moreover, in March, beliefs in conspiracy theories were directly and indirectly related (*via* perceived threat) to participants' implementation of preventive measures and vaccination intentions, and conspiracy beliefs in March also predicted preventive actions and vaccination intentions in July. The authors also found a negative relation between conspiracy beliefs and mask wearing (Romer and Jamieson, 2021a). Also, individuals prone to conspiracy theories reported less adherence to government guidelines and less willingness to get tested for or vaccinated against the virus (Freeman et al., 2020). Similarly, Murphy et al. (2021) found high levels of conspiracy beliefs in groups of vaccine-hesitant and -resistant people. Winter et al. (2021) also found that conspiracy mentality was negatively related to vaccination intentions. Hornsey et al. (2021) found in their cross-cultural study that beliefs in COVID-19 conspiracy theories were associated with greater self-related concerns but lower other-related concerns. In addition, conspiracy mentality in wave 1 predicted an aversion to COVID-19 vaccination in wave 2 (about 3 months later), and this effect was mediated by a lower importance of concern for others compared to self. In contrast, Bruder and Kunert (2022) reported mixed results: conspiracy mentality showed a negative association with contact-related preventive behavior but no relation to hygiene-related preventive behavior. Juanchich et al. (2021) also found mixed results in the United Kingdom: COVID-19 conspiracy believers did not differ from non-believers regarding the likelihood to follow hygiene- and contact-related preventive behaviors, but they were more reluctant to get tested and vaccinated and they also reported a lower willingness to install the official contact-tracing app by the National Health System. We hypothesized:

**H5:** Conspiracy mentality is negatively related to people's motivation to comply with all seven COVID-19 measures.

#### Health Locus of Control

Locus of control is a personality construct describing the extent to which a person believes that he or she is in control of events in life (Rotter, 1966; Kovaleva et al., 2012). The health locus of control (HLOC) describes control beliefs in health contexts. Individuals referred to as “health-internals” are thought to believe that they are in control of their health and that it is possible to become healthy or remain ill through one's behavior. “Health-externals” have the general expectation that they have no or only little influence on factors that determine their health (Wallston and Wallston, 1981). HLOC has an impact on diverse health behaviors. Specifically, health internals are more likely to show health-promoting behaviors such as taking care of dental hygiene, quitting smoking, losing weight, getting a flu vaccination, and using a seatbelt (Wallston and Wallston, 1978). There are also associations between HLOC and willingness to use mobile health apps and online trackers of health-specific information (Bennett et al., 2017). Mixed results have been found to date with respect to COVID-19 measures. For example, Berg and Lin (2020) reported that only one of several dimensions of their external HLOC instrument was positively related to the likelihood of engaging in COVID-19 preventive behaviors, whereas internal HLOC showed no association. In contrast, Murphy et al. (2021) found that the internal locus of control was stronger among vaccine skeptics. In principle, there are nonetheless arguments for both forms of HLOC as to why they might be positively related to compliance with protective measures, albeit from a different belief perspective. However, as the results are still too sparse with regard to the pandemic and corresponding measures, our exploratory (i.e., undirected) hypothesis was:

**H6:** Internal and external HLOC are related to people's motivation to comply with all seven COVID-19 measures.

#### General Affect

Finally, the experience of affect is inherent in each individual as a dispositional general tendency (Fox and Spector, 2000) and is substantially stable (Watson and Walker, 1996). There is also evidence for a link between affect and health behavior. Indeed, several studies have shown a positive association between health-related behavior and positive affect but a negative relation to negative affect, but most studies to date have focused on state affect (for reviews, see Pressman and Cohen, 2005; Sirois et al., 2015). To our knowledge, general (trait) affect has not yet been associated with adherence to COVID-19 measures, but given the relevance of affect for health behavior in general, we tested the following exploratory hypothesis:

**H7:** General positive and negative affect are related to people's motivation to comply with all seven COVID-19 measures.

## Methods

### Participants and Procedure

Recruitment was *via* convenience sampling. The link to the study was distributed through social media platforms including Instagram, Facebook, WhatsApp, and LinkedIn, as well as through a survey platform of a German journal (Psychologie Heute). At the beginning of the study, participants were informed that the data of this study will be used for research purposes and that all data would be collected anonymously. Thus, no identifying information was collected. Participants who prematurely stopped the survey were not included in the analyses and all of their data were deleted from the dataset. Informed consent to participate in this study was provided by clicking a corresponding box, and participation was voluntary in all cases. The study ran from 10 November 2020 to 29 December 2020.

A total of 1,026 German-speaking participants took part in this online study. Thirteen people were excluded because of early termination, lack of consent, or because they were under 18. In addition, those who reported “diverse” as their gender were excluded (*n* = 6) because this subgroup was too small to be reasonably integrated into gender-based analyses. The final sample thus consisted of 1,007 participants, 793 female, and 214 male, with a mean age of *M* = 37.74 years (*SD* = 13.86). The most frequently named educational qualification was a master's degree (*n* = 366), followed by a bachelor's degree (*n* = 215), a higher education entrance qualification (*n* = 194), a completed vocational training (*n* = 155), a secondary school diploma (*n* = 64), a main school diploma (*n* = 11), and no school diploma (*n* = 2).

After the participants were informed about the study content and gave their consent, they provided demographic information, including age, gender, and education level. They were then asked about their motivation to comply with seven COVID-19 measures (distance rule, hygiene rules, face mask rule, use of a contact-tracing app, sharing one's infection status *via* a contact-tracing app, reducing physical contacts, and getting vaccinated). Then they reported their perceived severity of and vulnerability to a coronavirus infection, general personality (Big Five and Dark Triad), conspiracy mentality, HLOC, and general affect.

### Measures

#### Motivation to Comply With COVID-19 Protective Measures

Participants indicated their motivation to comply with COVID-19 measures by using a 6-point rating scale ranging from 1 (*very unmotivated*) to 6 (*very motivated*). Items and corresponding statistics are presented in Table 1.

**Table 1:** Descriptive and inferential statistics for participants' motivation to comply with COVID-19 measures.

**Motivation to comply with COVID-19 measure**	***M***	***SD***	***t*** **(1006)**	***p***	***d***
How motivated are you to comply with the distance rule, i.e., to keep a physical distance of at least 1.5 m from other people outside your household?	5.10^a^	1.06	48.03	<0.001	1.51
How motivated are you to comply with the hygiene rules, i.e., proper coughing and sneezing and thorough hand washing?	5.48^b^	0.86	73.12	<0.001	2.30
How motivated are you to comply with the face mask rule, i.e., wearing mouth-to-nose coverings in all appropriately designated areas of public life?	5.24^c^	1.29	42.79	<0.001	1.35
How motivated are you to use the contact-tracing app, that is, installing and activating the app on your smartphone?	4.30^d^	1.92	13.21	<0.001	0.42
If you were to become infected with the coronavirus, how motivated would you be to voluntarily report your infection status in the contact-tracing app so that others can be warned after critical contact with you?	5.03^a^	1.63	29.85	<0.001	0.94
How motivated are you to reduce your physical contacts with others as much as possible, i.e., to avoid meeting others outside your household?	4.20^d^	1.31	17.04	<0.001	0.54
Given an officially approved vaccine against the coronavirus, how motivated would you be to get yourself vaccinated?	4.27^d^	1.67	14.69	<0.001	0.46

*All items used a response scale of 1 (very unmotivated) to 6 (very motivated). One-sample t-tests were computed to examine whether means differed from the scale's midpoint of 3.5. Mean values with different superscripts (a-d) indicate statistically significant differences between protective measures (Bonferroni-adjusted significance level, all ps ≤ 0.001). Effect size d represents small (d = 0.20), medium (d = 0.50) or large (d = 0.80) effects according to Cohen (1992)*.

#### Risk Perception

Items from Kaspar (2020) were used to assess the perceived severity of a coronavirus infection (e.g., “If I became infected with the coronavirus, it would have a strong negative effect on my health,” α = 0.91). Items measuring perceived vulnerability were adapted (cf. Kaspar, 2020) and linked to the German AHA-rules (e.g., “I have a significantly increased risk of a coronavirus infection, if I do not follow the AHA-rules (keep distance, follow hygiene measures, wear a face mask) to contain the coronavirus pandemic,” α = 0.83). Both constructs were measured with three items, each with a 7-point scale (1 = *disagree completely*, 7 = *agree completely*).

#### Big Five Traits

Big Five personality traits were assessed *via* the BFI-K (Rammstedt and John, 2005). This is a standardized and economical instrument for applied settings with good psychometric properties. Its factorial structure has been validated in both homogeneous student samples and larger heterogeneous samples (Kovaleva et al., 2013). The instrument comprises 21 items (1 = *disagree strongly*, 5 = *agree strongly*) measuring extraversion (α = 0.82, e.g., “I get out of myself, I am sociable”), neuroticism (α = 0.80, e.g., “I get depressed easily, dejected”), agreeableness (α = 0.63, e.g., “I trust others easily, believe in the good in people”), conscientiousness (α = 0.67, e.g., “I complete tasks thoroughly”), and openness to experience (α = 0.73, e.g., “I am interested in many things”). Personality traits are measured by four to five items each.

#### Dark Triad Traits

The German Short Dark Triad instrument (Malesza et al., 2019) was used to measure Machiavellianism (e.g., “I like to use clever manipulation to get my way,” α = 0.77), narcissism (e.g., “I know that I am special because everyone keeps telling me so,” α = 0.72), and psychopathy (e.g., “I like to get revenge on authorities,” α = 0.69). In total, 27 items (nine per trait) were rated on a 5-point scale (1 = *disagree strongly*, 5 = *agree strongly*). The factorial structure and validity of the instrument have been confirmed for the German version (Malesza et al., 2019) and its English original (Jones and Paulhus, 2014).

#### Conspiracy Mentality

To assess participants' conspiracy mentality, the Conspiracy Mentality Questionnaire (Bruder et al., 2013) was used. This instrument comprises five items (α = 0.86) and a response scale ranging from 0% (*certainly not*) to 100% (*certain*). Participants were asked to indicate their agreement with conspiracy ideas, such as “I think that many very important things happen in the world, which the public is never informed about” and “I think that politicians usually do not tell us the true motives for their decisions.” Factorial structure, test–retest–reliability, and validity of this one-dimensional construct have been demonstrated (Bruder et al., 2013).

#### Health Locos of Control (HLOC)

A questionnaire to assess health-related control beliefs with good psychometric properties was used (Ferring, 2003). This instrument includes five items on internal health control beliefs (e.g., “If you take care of yourself, you will stay healthy,” α = 0.78) and five items on external health control beliefs (e.g., “Good health is largely a matter of luck,” α = 0.84). The rating scale ranges from 1 (*very wrong*) to 6 (*very true*).

#### General Affect

We used the German version of the Positive and Negative Affective Schedule (PANAS) developed by Krohne et al. (1996). This instrument measures general (trait) and context-free positive affect (10 items, α = 0.83; e.g., interested, excited, active) and negative affect (10 items, α = 0.86; e.g., nervous, scared, distressed) by emotion-laden adjectives. Participants were asked to rate how they generally feel this way (cf. Watson et al., 1988) on a 5-point scale ranging from 1 (*not at all*) to 5 (*extremely*).

### Data Analysis

First, intercorrelations between the independent variables of the regression models were calculated to assess construct validity and identify critical construct overlap. Second, bivariate correlations between independent and dependent variables of the regression models were calculated as a reference for subsequent results of the regression models. Third, a multiple regression analysis was calculated for each of the seven protective measures. In this context, statistical assumptions being relevant were tested (cf. Poole and O'Farrell, 1971), and bootstrapping was used for inferential tests, as routinely suggested (cf. Hayes and Cai, 2007). Fourth, one-sample *t*-tests were calculated to test whether the mean motivation to comply with each measure deviated from the scale's midpoint to draw conclusions about above- or below-average compliance. Fifth, a repeated measures ANOVA (Greenhouse-Geisser applied) and Bonferroni-adjusted pairwise comparisons were calculated. Finally, a multiple regression analysis with conspiracy mentality as the dependent variable was calculated on an *ad hoc* basis for exploratory reasons.

## Results

As shown in Table 2, intercorrelations between independent variables of the multiple regression models were low overall with few notable exceptions: participants' age and their perceived severity of a coronavirus infection were positively related (*r* = 0.34). The Big Five traits showed low intercorrelations, with the highest correlation being between extraversion and neuroticism (*r* = −0.30). Dark Triad traits were positively correlated, with the highest correlation being between Machiavellianism and subclinical psychopathy (*r* = 0.48). Conspiracy mentality showed the highest (negative) correlation with perceived vulnerability to infection (*r* = −0.27). Internal and external HLOC were negatively correlated (*r* = −0.50). Finally, negative and positive trait affect were negatively correlated (*r* = −0.26). Intercorrelations with exact *p*-values are presented in the Supplementary Material.

**Table 2:** Bivariate correlations between independent variables of the regression models.

**Independent variable**	**1**.	**2**.	**3**.	**4**.	**5**.	**6**.	**7**.	**8**.	**9**.	**10**.	**11**.	**12**.	**13**.	**14**.	**15**.	**16**.
1. Age																
2. Gender	−0.14^***^															
3. Severity of infection	0.34^***^	0.04														
4. Vulnerability to infection	0.07^*^	0.04	0.46^***^													
5. Extraversion	−0.02	0.08^**^	−0.08^**^	0.02												
6. Neuroticism	−0.20^***^	0.21^***^	0.13^***^	0.08^*^	−0.30^***^											
7. Agreeableness	−0.04	0.07^*^	−0.09^**^	0.03	0.17^***^	−0.13^***^										
8. Conscientiousness	0.07^*^	0.15^***^	−0.04	0.08^**^	0.21^***^	−0.14^***^	0.09^**^									
9. Openness	0.15^***^	0.03	0.13^***^	0.03	0.11^***^	0.02	0.09^**^	0.08^**^								
10. Machiavellianism	−0.08^*^	−0.17^***^	0.04	0.01	−0.06	0.08^*^	−0.37^***^	−0.12^***^	−0.12^***^							
11. Narcissism	0.05	−0.13^***^	0.01	−0.01	0.44^***^	−0.30^***^	−0.07^*^	0.11^***^	0.16^***^	0.24^***^						
12. Psychopathy	−0.08^*^	−0.22^***^	−0.05	−0.08^*^	0.07^*^	−0.01	−0.39^***^	−0.17^***^	−0.01	0.48^***^	0.36^***^					
13. Conspiracy mentality	−0.08^*^	0.06	−0.16^***^	−0.27^***^	0.02	0.03	−0.06	0.08^*^	−0.04	0.21^***^	0.07^*^	0.21^***^				
14. Internal HLOC	−0.05	−0.09^**^	−0.08^*^	0.03	0.17^***^	−0.17^***^	0.09^**^	0.21^***^	0.07^*^	0.03	0.17^***^	−0.03	0.14^***^			
15. External HLOC	0.10^**^	0.11^***^	0.16^***^	0.06	−0.15^***^	0.16^***^	−0.05	−0.15^***^	−0.00	0.08^**^	−0.09^**^	0.02	−0.02	−0.50^***^		
16. Positive affect	0.09^**^	−0.02	−0.05	0.06	0.45^***^	−0.43^***^	0.14^***^	0.42^***^	0.16^***^	−0.06^*^	0.40^***^	−0.01	0.04	0.29^***^	−0.18^***^	
17. Negative affect	−0.08^*^	0.03	0.13^***^	0.04	−0.22^***^	0.56^***^	−0.20^***^	−0.19^***^	−0.01	0.17^***^	−0.11^***^	0.19^***^	0.09^**^	−0.12^***^	0.14^***^	−0.26^***^

*Gender was dummy-coded (0 = male, 1 = female),*

* *p < 0.05,*

** *p < 0.01,*

*** *p < 0.001*.

As shown in Table 3, apart from being statistically significant in almost half of the cases (44.5%), bivariate correlations between independent and dependent variables of the multiple regression models were low overall. Perceived severity of and vulnerability to a coronavirus infection showed positive correlations with all COVID-19 measures, although correlations between perceived vulnerability and the seven protective measures were consistently higher. Age, gender, the Big Five and Dark Triad traits, HLOC, and general affect showed low to zero correlations with most measures. In contrast, conspiracy mentality was consistently negatively correlated with all measures.

**Table 3:** Bivariate correlations between independent and dependent variables of the regression models.

**Independent variables**	**Complying with** **distance rule**		**Complying with** **hygiene rules**		**Wearing a** **face mask**		**Using the** **contact-tracing app**		**Sharing one's infection** **status** ***via*** **app**		**Reducing physical** **contacts**		**Getting** **vaccinated**	
	***r***	***p***	***r***	***p***	***r***	***p***	***r***	***p***	***r***	***p***	***r***	***p***	***r***	***p***
Age	0.20	<0.001	0.06	0.046	0.07	0.020	0.03	0.412	−0.05	0.142	0.23	<0.001	0.08	0.011
Gender	0.10	0.002	0.14	<0.001	0.01	0.674	−0.03	0.350	−0.03	0.416	0.02	0.460	−0.16	<0.001
Severity of infection	0.33	<0.001	0.21	<0.001	0.30	<0.001	0.17	<0.001	0.19	<0.001	0.37	<0.001	0.27	<0.001
Vulnerability to infection	0.50	<0.001	0.31	<0.001	0.56	<0.001	0.34	<0.001	0.41	<0.001	0.50	<0.001	0.43	<0.001
Extraversion	−0.05	0.142	0.05	0.139	0.02	0.501	0.04	0.273	0.04	0.191	−0.10	0.002	0.00	0.938
Neuroticism	0.02	0.593	−0.03	0.297	0.01	0.761	−0.06	0.072	−0.01	0.663	0.01	0.763	−0.06	0.068
Agreeableness	−0.03	0.379	0.01	0.699	0.03	0.414	0.10	0.001	0.08	0.010	0.01	0.727	0.03	0.410
Conscientiousness	0.07	0.018	0.17	<0.001	0.01	0.759	0.04	0.234	0.03	0.437	0.05	0.153	−0.06	0.075
Openness	−0.00	0.985	0.04	0.240	−0.02	0.552	−0.05	0.107	−0.03	0.306	0.00	0.967	−0.02	0.557
Machiavellianism	−0.05	0.127	−0.07	0.023	−0.04	0.233	−0.08	0.008	−0.10	0.001	−0.08	0.016	0.01	0.885
Narcissism	−0.05	0.094	0.00	0.897	−0.02	0.535	−0.00	0.975	0.00	0.973	−0.12	<0.001	0.02	0.572
Psychopathy	−0.15	<0.001	−0.13	<0.001	−0.11	0.001	−0.14	<0.001	−0.10	0.002	−0.18	<0.001	−0.03	0.305
Conspiracy mentality	−0.24	<0.001	−0.12	<0.001	−0.31	<0.001	−0.37	<0.001	−0.34	<0.001	−0.31	<0.001	−0.40	<0.001
Internal HLOC	−0.02	0.584	0.04	0.200	−0.06	0.058	−0.04	0.242	−0.03	0.397	−0.07	0.032	−0.09	0.007
External HLOC	0.07	0.025	0.05	0.102	0.09	0.004	0.03	0.427	0.02	0.641	0.10	0.002	0.05	0.094
Positive affect	0.06	0.064	0.12	<0.001	0.01	0.759	0.03	0.335	0.03	0.408	0.00	0.903	0.02	0.470
Negative affect	−0.01	0.833	−0.09	0.003	−0.03	0.370	−0.08	0.010	−0.04	0.182	−0.03	0.374	−0.01	0.842

*Gender was dummy-coded (0 = male, 1 = female)*.

The multiple regression analyses revealed that the joint contribution of all independent variables of the research model (see Figure 1) explained a significant amount of variance in participants' motivation to comply with the COVID-19 protective measures. However, explained variance ranged between 16% for hygiene rules and 35% for face masks and reduction of physical contacts. The analysis of the relevance of the individual factors showed the following pattern of results.

As shown in Table 4, participants' age (H1a) was positively related to their motivation to comply with the distance rule and to reduce physical contacts but it was negatively related to the motivation to share one's (positive) infection status *via* the contact-tracing app. Women, compared to men (H1b), indicated higher motivation to comply with the distance and hygiene rules, but lower motivation to get vaccinated. The perceived severity of a coronavirus infection (H2) was positively related to participants' motivation to comply with hygiene rules, to reduce physical contacts, and to get vaccinated. Perceived vulnerability to a coronavirus infection (H2) showed a positive relation to all COVID-19 measures and it was the most relevant factor regarding all but one measure, as indicated by the standardized regression coefficients. This result corresponds to the pattern of bivariate correlations shown in Table 3. Overall, the Big Five personality traits (H3) showed only a few significant relations to the motivation to comply with the measures: extraversion was negatively related to physical contact reduction. Neuroticism was negatively related to the motivation to get vaccinated. Agreeableness was negatively related to the distance rule. Conscientiousness was positively related to the hygiene rules. Openness to experience was negatively related to both using the national contact-tracing app and the willingness to share one's infection status *via* this app. Similarly, the Dark Triad traits (H4) were only weakly related to the measures. Machiavellianism was negatively related to the willingness to share one's infection status *via* the contact-tracing app. Subclinical narcissism did not show a significant relation to the measures. Subclinical psychopathy showed a negative relation to the distance rule and physical contact reduction. In contrast, conspiracy mentality (H5) was a strong factor that was negatively related to all measures, except hygiene rules. Internal HLOC (H6) showed a negative relation to participants' motivation to get vaccinated, external HLOC (H6) showed a positive relation to the hygiene rules. Positive and negative trait affect (H7) showed no significant relation to the measures.

**Table 4:** Results of the multiple regression analyses for the seven COVID-19 protective measures.

**Independent variable**	**Complying with** **distance rule**		**Complying with** **hygiene rules**		**Wearing a** **face mask**		**Using the** **contact-tracing app**		**Sharing one's** **infection status** ***via*** **app**		**Reducing physical** **contacts**		**Getting** **vaccinated**	
	**β**	***p***	**β**	**5 *p***	**β**	***p***	**β**	***p***	**β**	***p***	**β**	***p***	**β**	***p***
Age	0.14	<0.001	−0.01	0.676	−0.00	0.978	−0.04	0.220	−0.12	<0.001	0.14	<0.001	−0.03	0.366
Gender	0.10	0.001	0.10	0.005	−0.01	0.774	−0.04	0.216	−0.06	0.064	0.02	0.569	−0.14	<0.001
Severity of infection	0.06	0.061	0.11	0.006	0.05	0.161	0.04	0.239	0.06	0.068	0.11	0.001	0.11	0.002
Vulnerability to infection	0.43	<0.001	0.23	<0.001	0.50	<0.001	0.24	<0.001	0.33	<0.001	0.39	<0.001	0.31	<0.001
Extraversion	−0.06	0.063	−0.01	0.790	0.03	0.351	0.01	0.735	0.02	0.550	−0.08	0.021	0.01	0.777
Neuroticism	−0.02	0.606	−0.02	0.556	−0.03	0.492	−0.04	0.266	−0.03	0.511	−0.03	0.327	−0.08	0.020
Agreeableness	−0.07	0.028	−0.06	0.080	−0.02	0.624	0.06	0.072	0.03	0.340	−0.03	0.425	0.04	0.235
Conscientiousness	0.01	0.860	0.11	0.002	−0.02	0.579	0.04	0.248	0.02	0.534	0.02	0.541	−0.03	0.364
Openness	−0.05	0.097	−0.00	0.941	−0.04	0.154	−0.08	0.009	−0.06	0.049	−0.03	0.248	−0.04	0.188
Machiavellianism	0.00	0.896	−0.03	0.400	−0.00	0.981	0.00	0.920	−0.07	0.039	0.01	0.831	0.04	0.214
Narcissism	−0.01	0.715	0.00	0.987	0.01	0.723	0.04	0.245	0.04	0.308	−0.07	0.063	0.00	0.949
Psychopathy	−0.08	0.031	−0.06	0.183	−0.05	0.125	−0.06	0.121	−0.01	0.839	−0.08	0.017	0.00	0.967
Conspiracy mentality	−0.10	0.001	−0.04	0.206	−0.15	<0.001	−0.29	<0.001	−0.23	<0.001	−0.15	<0.001	−0.29	<0.001
Internal HLOC	0.01	0.784	0.05	0.176	−0.03	0.289	−0.02	0.569	−0.02	0.603	−0.02	0.482	−0.07	0.031
External HLOC	0.02	0.566	0.08	0.016	0.04	0.136	0.02	0.610	0.01	0.752	0.03	0.371	0.00	0.986
Positive affect	0.07	0.051	0.05	0.237	−0.01	0.706	−0.02	0.691	−0.01	0.794	0.03	0.324	0.03	0.456
Negative affect	−0.00	0.970	−0.07	0.091	−0.03	0.415	−0.02	0.592	−0.01	0.811	−0.02	0.458	0.04	0.265
*R^2^* ($R_{adjusted}^{2}$)	0.32 (0.31)	<0.001	0.16 (0.14)	<0.001	0.35 (0.34)	<0.001	0.22 (0.21)	<0.001	0.25 (0.24)	<0.001	0.35 (0.34)	<0.001	0.31 (0.30)	<0.001

*Gender was dummy-coded (0 = male, 1 = female), p-values are based on bootstrapping (10,000 iterations)*.

Finally, participants' motivation to comply with COVID-19 measures was relatively high (above the scales' midpoints), as indicated by one-sample *t*-tests, all *t*s_(1,006)_ ≥ 13.21, all *p*s < 0.001, all *d*s ≥ 0.42 (detailed results, see Table 1). However, an ANOVA for repeated measures, *F*_(4.20,4,224.18)_ = 237.320, *p* < 0.001, $\eta_{\text{p}}^{2}$ = 0.191, and subsequently computed Bonferroni-adjusted pairwise comparisons showed that motivation decreased from hygiene rules (first rank) to face mask rule (second rank) to social distancing and willingness to share one's infection status *via* the contact-tracing app (shared third rank) to using the app, reducing physical contacts, and vaccination (shared fourth rank), see Table 1.

Given the robust negative relation between conspiracy mentality and the protective measures as well as increasing interest in studying conspiracy mentality in the context of the COVID-19 pandemic, we performed another multiple regression on an *ad hoc* basis for exploratory reasons. We included all of the independent variables from the previous regression models, except for conspiracy mentality, which served as the dependent variable this time. The regression model explained 19% of variance (*p* < 0.001). Perceived vulnerability to infection (β = −0.28, *p* < 0.001) showed a negative relation to conspiracy mentality. In contrast, conspiracy mentality was positively related to gender (dummy-coded: 0 = male, 1 = female; β = 0.12, *p* < 0.001), agreeableness (β = 0.07, *p* = 0.039), conscientiousness (β = 0.11, *p* = 0.002), Machiavellianism (β = 0.16, *p* < 0.001), subclinical psychopathy (β = 0.18, *p* < 0.001), internal HLOC (β = 0.17, *p* < 0.001), and negative affect (β = 0.08, *p* = 0.038), reflecting most of the bivariate correlations between conspiracy mentality and respective variables, with the exception of agreeableness. Participants' age, perceived severity of infection, extraversion, neuroticism, openness, narcissism, external HLOC, and positive affect were not significantly related to conspiracy mentality in the regression model.

## Discussion

In this study, we investigated associations between personal characteristics and motivation to comply with seven COVID-19 protective measures. The study aimed at filling an empirical gap in the young literature already published. Considering bivariate correlations and the results of the multiple regression analyses that examined the joint contribution of all personal characteristics simultaneously and their individual relevance, we will discuss the main findings, their implications for research and practice, as well as limitations in the following.

### The Role of Demographic Variables

As expected, age showed positive but small bivariate correlations with five of the seven protective measures, with the exception of using the contact-tracing app and sharing one's infection status through the app. In contrast, some associations disappeared when the joint contribution of all personal characteristics was examined simultaneously *via* multiple regression models. In this case, age was positively related to the motivation to comply with social distancing and reducing physical contacts, indicating that distancing measures are particularly important for seniors. Given their higher health risk, they might be more motivated to avoid contact with others to minimize their risk of infection. Also, larger social gatherings might play a greater role in younger individuals, decreasing their motivation in this regard. This is consistent with some previous findings showing that older adults reported higher compliance with COVID-19 protective measures (Brouard et al., 2020; Dohle et al., 2020; Zettler et al., 2022) and specifically engaging in physical distancing (Zettler et al., 2022). However, age was not associated with other preventive measures in the regression models, i.e., hygiene rules, wearing face masks, and vaccination readiness, although this would seem reasonable given the increased risk of the elderly to a potentially severe disease course (Jordan et al., 2020). In contrast to a non-significant bivariate correlation, higher age was significantly associated with lower motivation to share one's infection status *via* the contact-tracing app in the multiple regression model. Since sharing the infection status, as opposed to simply downloading the app, requires an active use and a certain familiarity with the app, the general lower willingness of older adults to use smartphone apps may come into play here (Cho, 2015). Older participants may also be more concerned about the security of their personal information.

In general, age effects may be even more pronounced when comparing young and very old individuals, but none of the previous studies in this field realized such a specific comparison. Although the present sample had an age range of 18–82 years (mean 38), only 1% of the participants were over 70 years old, representing a group being at higher risk of developing severe health consequences (cf. Wyper et al., 2020). Many of the samples studied have such restrictions in terms of the age range of participants, which could reduce the magnitude of the association between age and potentially related variables (cf. Mendoza and Mumford, 1987). In addition, the relevance of age in the context of COVID-19 measures may depend in part on the practical feasibility of each measure (e.g., availability and use of a contact-tracing app, vaccination for all or only for certain age groups, or social distancing in care facilities) and the median age of populations, which considerably varies across countries (cf. Pew Research Center, 2020). Consequently, future research should emphasize cross-national comparisons and context variables that may moderate the relevance of age to comply with COVID-19 measures.

The results regarding gender were identical for the bivariate correlations and the multiple regression analyses. In line with previous research on gender-related differences in compliance with COVID-19 measures (e.g., Abdelrahman, 2020; Brouard et al., 2020; Dohle et al., 2020; Zettler et al., 2022), women reported higher motivation to comply with social distancing and the hygiene rules. Accordingly, in a study comparing eight Western countries, Galasso et al. (2020) showed that women differed from men in having a higher “belief that COVID-19 represents a very serious health risk, in their agreement with restraining public health rules, and in their compliance with them” (p. 27,290). However, results were based on composite scores, and hence no differentiated picture across measures was presented. In the present study, motivation to comply with wearing face masks, reducing physical contact, using the contact-tracing app, and actively sharing one's infection status was unrelated to gender, indicating that gender differences are less relevant in this context. In contrast, men reported higher motivation to get vaccinated. This result is in line with meta-analytic findings according to which more men stated that they would get vaccinated. This phenomenon appears to be stable across countries and more pronounced in samples with health care workers (Zintel et al., 2022). However, the reasons for this finding are less clear and should be addressed by future research. One reason might be that even before the current COVID-19 pandemic and related vaccines, women typically reported more side effects after vaccinations than men (for a review, see Flanagan et al., 2017). Klugar et al. (2021) found that women reported an increased risk of side effects after either mRNA-based or viral vector-based COVID-19 vaccines.

These age- and gender-related results exemplarily show that different measures to combat the pandemic are strongly linked to sociodemographic variables, associated with varying levels of acceptance and different reasons for acceptance of measures. Consequently, public presentations of study results and associated recommendations for appropriate health behavior should consider individual differences in the subjective threat appraisal and the objective risk situation. Target group-specific approaches and education could increase the overall acceptance of the different measures across heterogeneous groups.

### The Role of Risk Perception

Perceived severity of coronavirus infection showed a positive but rather low bivariate correlation with all the seven protective measures. In contrast, multiple regression models showed a positive association between perceived severity of infection and participants' motivation to follow hygiene rules, reduce physical contact, and get vaccinated. In addition, perceived vulnerability to coronavirus infection showed a positive association with all COVID-19 measures at both the bivariate correlation level and the multiple regression level. Moreover, the perceived vulnerability was the most relevant factor regarding all measures except app use in the regression models. It is important to note that this pattern of results remarkably differs from what Kaspar (2020) previously reported regarding people's motivation for social distancing, app use, and sharing one's infection status *via* the app. While Kaspar (2020) used a regression model incorporating all core variables of the Protection Motivation Theory (Rogers, 1983) and some context-specific variables, the present model shifted the focus to context-independent personal characteristics. Therefore, the relevance of perceived severity and vulnerability must be interpreted in the context of the specific research model. With respect to the current and dynamic pandemic, it is also particularly important to consider the point in time a study is conducted. In this case, perceived vulnerability to infection was the most relevant factor, even outweighing the severity of infection. However, this result could change with new variants of the virus. When this study was conducted, the now steadily expanding Omicron variant was not foreseeable. Since this variant appears to have higher transmissibility (Karim and Karim, 2021) but milder effects on health (Nealon and Cowling, 2022) than earlier variants, the role of perceived vulnerability to and severity of infection might change. For example, in the context of Protection Motivation Theory, Floyd et al. (2000) argued that “the threat-appraisal process is addressed first, since a threat must be perceived or identified before there can be an evaluation of the coping options” (p. 410). In other words, if an infection with a new virus variant is assessed as hardly or not at all severe, possible coping strategies such as social distancing or vaccination may no longer be considered at all, and associations/correlations between severity assessment and motivation to follow certain measures disappear. Such a potential moderation effect, which could be caused by different variants of the SARS-CoV-2 virus, has not yet been empirically investigated but seems very reasonable.

At the same time, individual vaccination status also significantly affects the perceived severity of a potential infection as the number of vaccinations received and their timing appear to play a significant role in possible infections and severe courses of disease (e.g., Abu-Raddad et al., 2021; Andrews et al., 2021; Doria-Rose et al., 2021). In this respect, the results of the present and previous studies may be subject to greater temporal changes than is the case in other health-related areas with more time-stable contextual factors (e.g., skin cancer screening or caries prophylaxis).

Nevertheless, three things are important: first, we should not make the mistake of underestimating the validity of previously reported results, although they may change over time. Future research should rather specifically identify the role of time-varying and time-invariant factors. Until then, it remains unclear whether perceived vulnerability and severity are more likely to be traits or states. Second, the population should be accurately informed and kept up to date with respect to actual virus transmissibility (and the measures that reduce it) because vulnerability to infection appears to be particularly relevant to intended adherence to COVID-19 protective measures. This requires ongoing outreach by scientific institutions and political bodies, guided by consistent communication criteria. Third, among all the constructs (traits) examined, the construct of perceived vulnerability is likely to be particularly sensitive to contextual factors. Future research models should therefore consider situational factors that might moderate the relationship between perceived vulnerability and compliance with COVID-19 measures. Corresponding factors could significantly increase the explanatory power of research models in the future, even though the explanatory power of the present model is already remarkable, at least with regard to some of the COVID-19 measures investigated here.

### The Role of Personality Traits

Contrary to our hypotheses, the present data suggest that the Big Five do not play a central role in the context of COVID-19 protective measures, neither in the multiple regression models nor at the level of bivariate correlations between traits and motivation to comply with measures. We found that extraversion was negatively related to the reduction of physical contacts at both the bivariate correlation level and the multiple regression level. This is consistent with the finding of a negative association between extraversion and general rule-following (Clark et al., 2020). However, extraversion showed no relationship to other measures in the present study, whereas other studies found positive relations between extraversion and motivation to wear a face mask and to follow hygiene rules (Aschwanden et al., 2021).

The level of reported neuroticism showed no significant bivariate correlation with any of the seven protective measures. However, we found that neuroticism was negatively associated with motivation to get vaccinated in the respective multiple regression model. In other studies, neuroticism has been associated with lower caution in avoiding COVID-19 (Aschwanden et al., 2021), but neuroticism was positively associated with social distancing (Abdelrahman, 2020) and willingness to reduce contacts by staying at home (Götz et al., 2020), which was not replicated in this study.

Agreeableness showed a positive bivariate correlation with using the contact-tracing app and sharing one's infection status through this app. This result is not surprising as this app does not help individual users to actively prevent infection, but its use is important to fight the pandemic on a broad societal level. Thus, the use of this app indicates solidarity, which reflects facets of agreeableness, namely the motivation to compromise and cooperate. In the multiple regression models, however, agreeableness was negatively and exclusively related to the distance rule. Interestingly, agreeableness was positively related to physical distancing in another study using a regression model that includes a different set of independent variables (Aschwanden et al., 2021).

In contrast, conscientiousness was positively related to the hygiene rules in this study and the study of Aschwanden et al. (2021). This positive relation was present at both the bivariate correlation level and the multiple regression level. In addition, conscientiousness showed a positive but small bivariate correlation with participants' motivation to comply with the distance rule. It is not implausible that permanently maintaining a minimum distance from other people is associated with more pronounced conscientiousness.

Similar to neuroticism, the degree of reported openness to experience showed no significant bivariate correlation with any of the seven protective measures. However, we found that openness was negatively related to both using the national contact-tracing app and the willingness to share one's infection status *via* this app in the multiple regression models. This is a novel but an unexpected result as these COVID-19 measures were not captured by other studies on personality. It seems counterintuitive that general openness to new experiences is associated with less openness to technology-based interventions to address the pandemic. It is important to reiterate that using the app and sharing one's infection status *via* the app does not create a preventive effect for users, which might explain this result. However, these measures are helpful for other people and the fight against the pandemic on a broader societal level. In general, technology is likely to play a central role in controlling future endemic and pandemic scenarios, and the app use is not a new approach, as it has already been demonstrated in the context of the Ebola epidemic in West Africa between 2014 and 2015 (for a review, see Tom-Aba et al., 2018). Consequently, and contrary to the finding here, it appears necessary to promote a certain “true” openness in society to such technological measures by adequately addressing concerns about the privacy of one's data and the usefulness of the technology.

Indeed, besides the trait openness, general trust in app providers and perceived vulnerability to data misuse seem to be significant determinants of people's willingness to use a contact-tracing app and to share one's infection status (Kaspar, 2020), while perceived usefulness mainly predicts one's intention to use technology according to the well-established technology-acceptance model (Venkatesh and Bala, 2008). In summary, the current literature, including the present study, paints a very mixed picture regarding the Big Five in the context of COVID-19 protective measures. Some differences between studies could be partially attributed to different research models and divergent study groups. Overall, however, the Big Five appear to play a relatively weak role when several other factors are considered simultaneously.

Similarly, Dark Triad characteristics were also only weakly related to the measures when included simultaneously with other factors in the multiple regression models. In this case, Machiavellianism was negatively related to the willingness to share one's infection status *via* the contact-tracing app. Subclinical narcissism did not show a significant relation to the measures. Subclinical psychopathy showed a negative relation to the distance rule and physical contact reduction. At the level of bivariate correlations, there were more correlations with the protective measures, although these were generally very small, albeit statistically significant. It is noteworthy that in the case of a significant relation between Dark Triad traits and protective measures (regardless of whether in the context of multiple or bivariate regressions), this always turned out to be negative for all three “dark” traits. In particular, subclinical psychopathy showed negative correlations with all measures except vaccination readiness. In this respect, it seems important to include these personality traits in future studies and to consider them in practice, as they might be inhibiting factors for the success of COVID-19 measures.

### The Role of Conspiracy Mentality

In contrast to personality traits, conspiracy mentality was a strong factor that was negatively correlated with all measures and showed negative associations with all measures (except hygiene rules) in the multiple regression models. This result supports some but not all of the mixed results found in other studies (Freeman et al., 2020; Allington et al., 2021; Bruder and Kunert, 2022), and it is particularly important because several specific conspiracy theories and beliefs have already emerged in connection with the COVID-19 pandemic (cf. Juanchich et al., 2021). Moreover, the sample studied here had a strong academic background, with 58% of participants reporting at least a bachelor's degree. Nevertheless, even in such an academic sample, the level of conspiracy beliefs seems to play a truly significant role in the acceptance of COVID-19 measures. A look at the concrete operationalization of the construct in the present study shows that trust in political actors and in public communication plays a key role (e.g., “I think that many very important things happen in the world, which the public is never informed about” and “I think that politicians usually do not tell us the true motives for their decisions”).

In addition, we conducted an exploratory multiple regression analysis in which conspiracy mentality served as the dependent variable: conspiracy mentality was negatively related to perceived vulnerability to infection but not to the perceived severity of infection. Similarly, Romer and Jamieson (2020) found in their survey that conspiracy beliefs were inversely related to the perceived possibility that the respondents or someone in their families will become infected with the coronavirus. Accordingly, with an increasingly pronounced conspiracy mentality, the assessment that one can be infected with the virus decreases. This is remarkable because the virus does not care about the mindset the individual host has. At the conceptual level, this result shows the importance of distinguishing between the concepts of vulnerability to and severity of infection, which mirrors the different threat assessment components in protection motivation theory (cf. Rogers, 1983). In addition, conspiracy mentality was higher in women, in participants with pronounced beliefs that they are in control of their health (internal HLOC), and in participants who experienced high negative affect. Interestingly, conspiracy mentality was also positively related to Machiavellianism and subclinical psychopathy, replicating findings of other studies focusing on different populations such as U.K. citizens (Hughes and Machan, 2021). Surprisingly, conspiracy mentality was also positively related to agreeableness and conscientiousness in the regression model. While the positive association with conscientiousness does not seem implausible, since the tendency to engage with and defend alternative theories of system interrelations and processes requires a certain persistence, the positive association with agreeableness (including trust in others and belief in the goodness of people) seems unexpected. Since the corresponding bivariate correlation was not significant and also showed a negative sign, this finding seems to be the result of the regression model in which special suppression effects occurred.

Given these results, we may conclude that transparent communication of political decisions is required, which ideally should be evidence-based to counteract the significant role of conspiracy beliefs in the COVID-19 pandemic. In this context, the communication of scientific research practices and results in a way that is appropriate to the target audience plays also an important role. Rational and ridiculing arguments were found to be effective in reducing conspiracy beliefs (Orosz et al., 2016). Importantly, Hornsey et al. (2018) have pointed out that their data from a large cross-cultural study suggest communication solutions rather than repetition of evidence to effectively address vaccination skepticism. Furthermore, effective mechanisms should be installed to curb the rapid spread of misinformation in the sense of an “infodemic” (cf. Eysenbach, 2020). In principle, all media professionals have a special responsibility when it comes to the appropriate examination and dissemination of information that is important for the assessment of protective measures and associated behavioral tendencies. Indeed, Romer and Jamieson (2021b) found that the use of conservative media (e.g., Fox News) and social media (e.g., Facebook and Twitter) by U.S. residents was positively related to conspiracy beliefs related to the COVID-19 pandemic, whereas reliance on mainstream print (e.g., New York Times and Washington Post) predicted a decrease in such conspiracy beliefs. However, it should not be overlooked that individual characteristics, such as tendencies toward analytical thinking and spirituality (Gligorić et al., 2021), as well as the educational level (Georgiou et al., 2019), strongly interact with conspiracy beliefs, and thus measures taken by policymakers, academics, and the media can hardly lead to a reduction of conspiracy belief among all people.

### The Role of Health-Related Control Beliefs

Health-related control beliefs allow an estimation of the extent to which people consider their health a result of their actions (internal HLOC) or a result of external circumstances and chances that cannot be influenced (external HLOC). Overall, these variables showed a weak relation to compliance with COVID-19 measures in concert with all the other factors. At least, internal HLOC showed a negative bivariate correlation with reductions in physical contacts and vaccination readiness, as well as a negative relation to vaccination readiness in the multiple regression model. In a parallel study, Murphy et al. (2021) also found that the internal locus of control was stronger among vaccine skeptics. Apparently, people who are particularly convinced that they are in control of their health and that one can become healthy or stay sick through one's own behavior seem to be more opposed to the injection of a foreign substance. This appears consistent with one's control beliefs in that the production and effects of vaccines are beyond one's control. Nonetheless, this ignores the proven efficacy of licensed vaccines. However, it is important to note that at the start of this study (November 2020), no COVID-19 vaccine had been definitively approved for use in Germany, but results were already available demonstrating the vaccine's efficacy, and a vaccination start was officially envisioned (Bundesgesundheitsministerium, 2020). In this respect, there was no comprehensive experience with the vaccines at the time of the study, which may have affected the results.

In addition, we found that external HLOC was positively correlated with participants' motivation to comply with the distance rule, wear a face mask, and reduce physical contacts. In contrast, external HLOC showed only a positive relation to the hygiene rules in the multiple regression model. As people with high external HLOC have a general expectation that they have no or only little influence on factors that determine their health (Wallston and Wallston, 1981), they may be particularly devoted to those measures that they can obviously control themselves, such as proper coughing and sneezing and hand washing. Other measures such as the use of apps and vaccinations may be unrelated to external HLOC because these measures are based on technological and scientific progress, as well as concrete realizations over which individual users actually have no control.

### The Role of General Affect

The current and ongoing pandemic is a major strain on everyone and has been shown to negatively impact the emotional state of many people (e.g., Cao et al., 2020; Salari et al., 2020). Several studies have focused on state affect as a dependent variable in the context of the pandemic (e.g., Hardin et al., 2021). Some studies also included state affect as an independent variable, for example, to explain computer-mediated communication during the pandemic (Meier et al., 2021). However, the role of general (trait) affect has been widely neglected so far in the context of COVID-19 research. Based on the present results, we must conclude that trait affect is indeed not a relevant factor, at least as far as COVID-19 measures are concerned. Positive as well as negative trait affect did not show any significant relationship with the seven measures within the multiple regression model. Even at the level of bivariate correlations, only a few very small correlations were found. This result is nonetheless insightful because it seems that motivation to comply with various measures is less related to basic emotional factors than to cognitive factors (e.g., risk perception and conspiracy mentality).

### Limitations

Some limitations of the study should be noted. First, the data collected in this online study were self-report data, and social desirability could have potentially biased the responses. However, as shown by meta-analytic results, computerized surveys lead to significantly more reporting of socially undesirable behaviors (Gnambs and Kaspar, 2015).

Second, behavioral motivation was measured but not actual behavior. Motivation to follow COVID-19 measures does not necessarily mean that this behavior is performed. Several obstacles could jeopardize the transition from intention to action, such as technical problems with the contact-tracing app, temporary unavailability of face masks, problems organizing vaccination appointments, or social influence through the peer group. Nevertheless, from a research point of view, our approach was reasonable. In reference to the COVID-19 measures, monitoring actual behavior would mean to directly accompany and observe people in their daily life, which is difficult to realize. Additionally, given that “dark” traits such as conspiracy mentality, psychopathy, and Machiavellianism were negatively related to guideline adherence, it is questionable whether people in whom these traits are strongly pronounced would be willing to participate in a behavioral study. Consequently, self-selection among participants could occur, resulting in a sample of individuals who are highly motivated anyway and hence producing range restriction in collected data and biased statistical results. In addition, participants might be especially motivated to behave in socially desirable ways when they are overtly observed. Against this background, interviewing individuals seems to be the method of choice not only for reasons of research economics, which is evident in many current studies.

Third, the present research model was limited to personal characteristics. As stated above, motivation psychology emphasizes that a person's current motivation to strive for a certain goal is influenced by personal and situational factors (cf. Heckhausen and Heckhausen, 2006). Hence, it can be assumed that specific situational factors also explain a considerable proportion of the interpersonal variance in the motivation to comply with COVID-19 measures. These factors include, for example, the social environment, regional differences concerning applicable regulations, and job-related factors that could facilitate or impede the implementation of measures (e.g., social distancing, reducing physical contacts, and wearing a face mask).

Fourth, the present study focused on a comprehensive but simple research model, resulting in a multiple regression analysis. Many other studies that have been conducted previously or in parallel used the same or a similar methodological approach (e.g., Abdelrahman, 2020; Clark et al., 2020; Götz et al., 2020; Zajenkowski et al., 2020; Zettler et al., 2022), which does not specify relationships between model variables in terms of mediator and moderator variables. This makes sense at the beginning of such research, which has now been going on for just 2 years, especially since existing models do not cover all potentially relevant personal characteristics that may impact behavioral motivation regarding COVID-19 protective measures. As outlined in the introduction, relevant theories in the field of protection motivation (e.g., Protection Motivation Theory, Theory of Reasoned Action, and Theory of Planned Behavior) consider personal characteristics only as marginal variables. However, future studies may formulate and test more specific personality-oriented models based on the findings presented here and in other studies. At least, initial studies have examined indirect associations between conspiracy mentality and COVID-19-related preventive behaviors (e.g., Romer and Jamieson, 2020; Dijkstra, 2021), providing fruitful starting points.

Finally, the results are based on cross-sectional and correlational data. Therefore, no conclusion can be drawn about causal relationships between independent and dependent variables. This is true for most studies in the field, including those on indirect effects. Importantly, some authors propose double randomization to investigate a mediation hypothesis in terms of causal direction (e.g., Bullock et al., 2010; Green et al., 2010), leading to a sequence of experiments (cf. Kaspar and Cames, 2016). Here, participants are randomly assigned to levels of the independent variable to examine its effect on the observed mediator. Subsequently, participants are randomly assigned to different levels of the mediator to examine its effect on the observed dependent variable. However, in addition to the challenges of manipulating independent and mediator variables, ethical considerations might in principle militate against the manipulation of variables such as perceived vulnerability to infection and severity of infection.

## Conclusion

Participants reported a relatively high motivation to comply with COVID-19 measures, but significant differences between measures were apparent. Nevertheless, the average compliance was not maximal on any of the measures and there was considerable interpersonal variance. The present study underlines that personal characteristics have significant explanatory value for the motivation to comply with the measures.

The central regression models of the study show that basic personality traits in terms of the Big Five and Dark Triad played only a minor role. Nonetheless, their consideration in practice is important because the Dark Triad, for instance, consistently showed negative associations with protective measures, especially subclinical psychopathy at the level of bivariate correlations. The question therefore arises as to whether corresponding individual differences can be adequately taken into account at all by standardized measures. At the very least, against the background of these results, it becomes understandable why not all people follow the measures with the same willingness. One way to address this could be through programs that help people deal with the pandemic situation according to their individual personality profiles (cf. Michels et al., 2021). Such an approach may also be more effective than trying to achieve maximum compliance with the measures via politically driven top–down strategies.

Similar to personality traits, positive and negative trait affect and health-related control beliefs were of minor importance in the regression models, but they showed some bivariate correlations with the protective measures. Presumably, the role of situation-specific (state) affect is even more relevant, but this would still need to be shown. In principle, health-related control beliefs are an important factor in health-related education. However, their impact is likely to be overridden by more significant variables in the end, as the present results suggest.

The age and gender of the participants also revealed significant associations with some protective measures. These variables should therefore be taken into account when designing measures and communicating them in a way that is appropriate for the target group, especially since age and gender are not changeable by interventions, unlike some of the other variables studied here.

In contrast, the individual risk assessment (severity of and vulnerability to infection with SARS-CoV-2) was of remarkable relevance in the regression models and at the level of bivariate correlations. This result is not surprising against the background that a potential infection with the virus is the primary cause for all protective measures investigated. However, the result underscores how important it is for people to be empowered through appropriate education to make a realistic risk assessment.

In addition, conspiracy mentality was a strong and stable factor regarding protective measures in the multiple regression models and at the level of bivariate correlations. In all cases, the correlations with the protective measures were negative. Moreover, an exploratory analysis revealed that conspiracy mentality had several positive associations with other personal characteristics, but was negatively related to perceived vulnerability to infection. Given the particular importance of conspiracy beliefs in the public discourse of COVID-19 measures, it is clear that this variable cannot be viewed in isolation from other personal characteristics, but rather has a complex interrelationship with them. This makes it challenging to deal with it appropriately, and the important roles of communication strategies and the media in this context have already been pointed out.

Also worth mentioning is the fact that the willingness to use technology, which can effectively flank pandemic control, can be explained to a significant extent by personal characteristics. In this respect, an attempt should be made in this area to reconcile, as far as possible, individual motives for technology use and technology-related gratifications, as modeled in the context of the uses-and-gratification approach (cf. Katz et al., 1973).

In sum, the present results show that characteristics of an individual that are relevant to some measures may be irrelevant to other measures. It therefore seems necessary that differences in people's personal characteristics should be considered in the design of measures and the public communication of corresponding political decisions to support social acceptance and effectiveness of measures. In this context, cognitive variables, which can be addressed by communication and education directly, seem to play a more important role than general affect and relatively time-invariant personality traits. Protection motivation factors, in terms of perceived severity of and vulnerability to infection, and conspiracy mentality appear to be the major correlates of adopting protective behavior. In particular, when all independent variables were considered simultaneously in the regression model, the perceived vulnerability to infection and conspiracy mentality had the greatest significance across seven key pandemic measures. This finding complements previous research in which influencing variables were mostly examined in isolation or in smaller groups. From this, priorities for public communication and educational activities can be derived. The absolute motivation to comply with the measures also shows that hygiene rules and wearing face masks receive higher average agreement than more personally intrusive measures, such as physical contact restrictions and vaccinations, and thus seem to be easier to enforce. Thus, the results suggest that the potential of each measure should be estimated based on an individual profile of person-related variables that may be critical to the acceptance or rejection of the measure. The contribution of future research should lie in an appropriate formative evaluation strategy that examines both causal chains of impact and the extent and duration of actual compliance with measures.

## Data Availability Statement

The original contributions presented in the study are included in the article/Supplementary Material, further inquiries can be directed to the corresponding author.

## Ethics Statement

Ethical review and approval was not required for the study on human participants in accordance with the local legislation and institutional requirements. The patients/participants provided their written informed consent to participate in this study. All procedures performed in the study were in accordance with the ethical guidelines of the German Psychological Society (DGPs, http://www.dfg.de/foerderung/faq/geistes_sozialwissenschaften/index.html) and with the 1964 Helsinki Declaration.

## Author Contributions

KK administered the project, provided the resources, supervised the study, and revised the manuscript. KK and LN conceptualized the study, research methodology, analyzed the data, wrote the manuscript, and approved submission. LN collected the data. Both authors contributed to the article and approved the submitted version.

## Conflict of Interest

The authors declare that the research was conducted in the absence of any commercial or financial relationships that could be construed as a potential conflict of interest.

## Publisher's Note

All claims expressed in this article are solely those of the authors and do not necessarily represent those of their affiliated organizations, or those of the publisher, the editors and the reviewers. Any product that may be evaluated in this article, or claim that may be made by its manufacturer, is not guaranteed or endorsed by the publisher.
