# Supplementary material for: Personality and Motivation to Comply With COVID-19 Protective Measures in Germany
Source: Front Psychol. 2022 Jun 13;13:893881. doi: 10.3389/fpsyg.2022.893881 (PMC9234562; doi:10.3389/fpsyg.2022.893881)
Supplement: Supplementary file 1 [file Data_Sheet_1.PDF]

Supplementary Material

Table S: Bivariate correlations between independent variables of the regression models with exact *p*-values.

	**1.**		**2.**		**3.**		**4.**		**5.**		**6.**		**7.**		**8.**	
**Independent variable**	*r*	*p*	*r*	*p*	*r*	*p*	*r*	*p*	*r*	*p*	*r*	*p*	*r*	*p*	*r*	*p*
1. Age																
2. Gender	–.14	<.001														
3. Severity of infection	.34	<.001	.04	.234												
4. Vulnerability to infection	.07	.020	.04	.235	.46	<.001										
5. Extraversion	–.02	.588	.08	.009	–.08	.008	.02	.476								
6. Neuroticism	–.20	<.001	.21	<.001	.13	<.001	.08	.011	–.30	<.001						
7. Agreeableness	–.04	.246	.07	.030	–.09	.004	.03	.318	.17	<.001	–.13	<.001				
8. Conscientiousness	.07	.036	.15	<.001	–.04	.196	.08	.009	.21	<.001	–.14	<.001	.09	.005		
9. Openness	.15	<.001	.03	.352	.13	<.001	.03	.429	.11	<.001	.02	.563	.09	.004	.08	.009
10. Machiavellianism	–.08	.018	–.17	<.001	.04	.257	.01	.783	–.06	.055	.08	.012	–.37	<.001	–.12	<.001
11. Narcissism	.05	.129	–.13	<.001	.01	.690	–.01	.768	.44	<.001	–.30	<.001	–.07	.024	.11	<.001
12. Psychopathy	–.08	.018	–.22	<.001	–.05	.102	–.08	.014	.07	.040	–.01	.667	–.39	<.001	–.17	<.001
13. Conspiracy mentality	–.08	.014	.06	.062	–.16	<.001	–.27	<.001	.02	.537	.03	.338	–.06	.081	.08	.015
14. Internal HLOC	–.05	.153	–.09	.007	–.08	.010	.03	.359	.17	<.001	–.17	<.001	.09	.005	.21	<.001
15. External HLOC	.10	.002	.11	<.001	.16	<.001	.06	.081	–.15	<.001	.16	<.001	–.05	.154	–.15	<.001
16. Positive affect	.09	.003	–.02	.534	–.05	.132	.06	.069	.45	<.001	–.43	<.001	.14	<.001	.42	<.001
17. Negative affect	–.08	.015	.03	.415	.13	<.001	.04	.229	–.22	<.001	.56	<.001	–.20	<.001	–.19	<.001

	**9.**		**10.**		**11.**		**12.**		**13.**		**14.**		**15.**		**16.**	
	r	*p*	*r*	*p*	*r*	*p*	*r*	*p*	*r*	*p*	*r*	*p*	*r*	*p*	*r*	*p*
10. Machiavellianism	–.12	<.001														
11. Narcissism	.16	<.001	.24	<.001												
12. Psychopathy	–.01	.749	.48	<.001	.36	<.001										
13. Conspiracy mentality	–.04	.178	.21	<.001	.07	.018	.21	<.001								
14. Internal HLOC	.07	.038	.03	.378	.17	<.001	–.03	.428	.14	<.001						
15. External HLOC	–.00	.898	.08	.009	–.09	.003	.02	.572	–.02	.444	–.50	<.001				
16. Positive affect	.16	<.001	–.06	.043	.40	<.001	–.01	.758	.04	.176	.29	<.001	–.18	<.001		
17. Negative affect	–.01	.708	.17	<.001	–.11	<.001	.19	<.001	.09	.004	–.12	<.001	.14	<.001	–.26	<.001

Note: Gender was dummy-coded (0 = male, 1 = female).
